# Lunatics at Large

**Published:** 1908-09-26

**Authors:** 


### The Hospital

A Journal of

The Medical Sciences and Hospital Administration.

New Series. No. 81, Vol. III. [No. 1153, Vol. XLIV.] SATURDAY, SEPTEMBER 26, 1908.

### Lunatics at Large.

For some weeks past a correspondence has been appearing in the *Times* on the subject of escapes from lunatic asylums, and the practice of sending out from asylums walking parties of the insane. Epsom is specially blessed by the proximity of three or four large asylums of the London County Council, and many have been the complaints of what to outsiders is the gruesome spectacle of a walking party of forty or fifty lunatics, in their asylum dress, with the degraded and repulsive appearance that so many of them have, accompanied by attendants in uniform, parading the streets of the little country town, and, it is said, entering the shops, and even the public-houses. This, the inhabitants complain, is bad enough, but when some of the lunatics escape from these walking parties; when it is known that as many as fourteen escapes have taken place in a few months; and when a madman, stripped naked, is in the habit of jumping out of hedges at women and children who are passing by, the nuisance is become intolerable and must be abated. The allegations of fact are traversed by the authorities of the asylums. The number of escapes has been much exaggerated they say. The naked assailant of women and children is a myth. Visits to public-houses by the patients on leave are unknown, and appeals are made against prejudice and in favour of sympathy and indulgence towards beings afflicted with the greatest misfortune that humanity can suffer.

Weighing the two stories, it is easy to arrive at the real facts. Undoubtedly large walking parties of the insane do perambulate the streets of Epsom and the surrounding country roads and lanes. Undoubtedly escapes have taken place; undoubtedly the walking parties are a cause of uneasiness, of dread, and even of horror to the denizens of Epsom; and undoubtedly these feelings are intensified when it is known or suspected that patients have escaped from the asylums, and may be lurking in the neighbourhood, prepared to terrorise, to rob, to assault, nay, even to murder, unoffending passers by. It is small consolation to women and children to be told that the escaped lunatics are harmless. To the mind unaccustomed to the society of the insane, every lunatic is a potential homicide, and the very name "lunatic" is a word of terror. It seems a natural and reasonable request that under these circumstances the patients should be confined to the very ample area of the grounds of the asylums, and should not be permitted to remain a source of terror, or at least of uneasiness and repulsion, to the rest of the population. Such a request is the more reasonable since it appears from the letter of a doctor, formerly at the head of a large asylum, that the sending out of these walking parties is a matter of great anxiety to the superintendents, and is not very much desired by the patients themselves. Under these circumstances, since the practice is one which is desired by none of the parties interested, and is greatly deprecated by most of them, the question naturally arises, Why is it done at all? And the answer is a very curious one. It appears that it is done at the instance of the Lunacy Commissioners, who require, at each visitation of the asylum, an account of the number of patients "walking beyond the grounds," and insert these statistics in their published report. The asylum which can show a large percentage of patients thus privileged is by implication commended, and that which is wanting in this respect is by implication condemned. The Lunacy Commissioners appear to consider it their duty to enforce a reaction against the too stringent measures of restraint which were once employed in the treatment of the insane, and to cultivate among them a new delusion, that of making them believe they are being dealt with as if they were sane.

The practice is manifestly unintelligent and pernicious. "M.D." suggests that it should be abandoned, and that in its place should be substituted a practice of allowing carefully-selected curable cases to go out in small numbers and in private clothes, accompanied by attendants also in *mufti*. The suggestion has our hearty endorsement, and we would go further. A large number of inmates of asylums are every year restored to their friends and to the world at large on recovery. On one day they recover their full liberty, and go out and mingle with the world as sane persons. Yet the day before they would not be allowed beyond the asylum gates except as members of a large walking party in the charge of attendants. If a patient is sufficiently recovered to warrant a recommendation for his complete discharge, he is surely sufficiently recovered to be allowed outside the asylum walls by himself on parole. We have heretofore commented upon the rigid and unprogressive management of asylums for the insane, and it now appears that we must shift some of the responsibility for this state of things from the medical men in charge to the Commissioners themselves. Doubtless they are actuated by the best of motives, but upon their own showing they have not moved with the times. One of them is credited by "M.D." with the statement that they exist for the purpose of protecting the patients against ill treatment and coercive measures. This was all very well fifty years ago, and is even now needed in isolated cases of lunatics in private care and in care of their relatives; but with our public asylums, staffed by men and women brought up in the humane traditions of the modern treatment of insanity, and managed by enlightened and vigilant county and borough councillors, this particular function of the Commissioners is to a large extent superseded and unnecessary. The crying need of the present day in the treatment of insanity is not resistance to a spirit of coercion which no longer exists, but stimulation and encouragement of research into the nature and treatment of insanity. This is a function that might well be undertaken by the Commissioners in Lunacy; but instead of performing it, they fritter away their influence and lower their prestige by a routine adherence to obsolete methods. The Royal Commission on the Feeble-minded recommends that "the selection of men for the office of Commissioners . . . should have no relation to party claims or personal predilection, . . . the selection should rest entirely on individual suitability and should be biased by no other consideration whatever." Let us hope that this recommendation will be acted on in the future.

